# Time-varying living arrangements and suicide death in the general population sample: 14-year causal survival analysis via pooled logistic regression

**DOI:** 10.1017/S2045796024000325

**Published:** 2024-05-23

**Authors:** Z. Narita, T. Shinozaki, A. Goto, H. Hori, Y. Kim, H. C. Wilcox, M. Inoue, S. Tsugane, N. Sawada

**Affiliations:** 1 Department of Behavioral Medicine, National Institute of Mental Health, National Center of Neurology and Psychiatry, Kodaira, Tokyo, Japan; 2 Department of Information and Computer Technology, Faculty of Engineering, Tokyo University of Science, Katsushika-ku, Tokyo, Japan; 3 Department of Health Data Science, Graduate School of Data Science, Yokohama City University, Yokohama, Kanagawa, Japan; 4 Department of Mental Health, Johns Hopkins Bloomberg School of Public Health, Baltimore, MD, USA; 5 Division of Prevention, National Cancer Center Institute for Cancer Control, Chuo-ku, Tokyo, Japan; 6 Division of Cohort Research, National Cancer Center Institute for Cancer Control, Chuo-ku, Tokyo, Japan; 7 International University of Health and Welfare Graduate School of Public Health, Minato City, Tokyo, Japan

**Keywords:** all-cause mortality, causal inference, discrete-time hazard model, epidemiology, living alone, mental health, non-suicide death, public health, suicide prevention, survival analysis

## Abstract

**Aims:**

While past research suggested that living arrangements are associated with suicide death, no study has examined the impact of sustained living arrangements and the change in living arrangements. Also, previous survival analysis studies only reported a single hazard ratio (HR), whereas the actual HR may change over time. We aimed to address these limitations using causal inference approaches.

**Methods:**

Multi-point data from a general Japanese population sample were used. Participants reported their living arrangements twice within a 5-year time interval. After that, suicide death, non-suicide death and all-cause mortality were evaluated over 14 years. We used inverse probability weighted pooled logistic regression and cumulative incidence curve, evaluating the association of time-varying living arrangements with suicide death. We also studied non-suicide death and all-cause mortality to contextualize the association. Missing data for covariates were handled using random forest imputation.

**Results:**

A total of 86,749 participants were analysed, with a mean age (standard deviation) of 51.7 (7.90) at baseline. Of these, 306 died by suicide during the 14-year follow-up. Persistently living alone was associated with an increased risk of suicide death (risk difference [RD]: 1.1%, 95% confidence interval [CI]: 0.3–2.5%; risk ratio [RR]: 4.00, 95% CI: 1.83–7.41), non-suicide death (RD: 7.8%, 95% CI: 5.2–10.5%; RR: 1.56, 95% CI: 1.38–1.74) and all-cause mortality (RD: 8.7%, 95% CI: 6.2–11.3%; RR: 1.60, 95% CI: 1.42–1.79) at the end of the follow-up. The cumulative incidence curve showed that these associations were consistent throughout the follow-up. Across all types of mortality, the increased risk was smaller for those who started to live with someone and those who transitioned to living alone. The results remained robust in sensitivity analyses.

**Conclusions:**

Individuals who persistently live alone have an increased risk of suicide death as well as non-suicide death and all-cause mortality, whereas this impact is weaker for those who change their living arrangements.

## Introduction

Suicide represents a pressing public health concern globally, with approximately 700,000 deaths annually (World Health Organization, 2021a). It is vital to identify factors leading to suicidal behaviours to facilitate effective prevention efforts. Among various social factors, living alone has been suggested as important in the development of suicide (Nestadt, 2022). While the mechanism for this development remains unclear, living alone may increase a sense of thwarted belongingness, a crucial factor in the interpersonal theory of suicide (Van Orden *et al.*, 2010). Also, living alone may reduce the opportunities for intervention by others, contributing to the volitional phase in the integrated motivational–volitional model of suicide (O’Connor and Kirtley, 2018).

Previous studies have reinforced the evidence of an association between living alone and suicide death (Olfson *et al.*, 2022; Poudel-Tandukar *et al.*, 2011; Shaw *et al.*, 2021). A cohort study for the Japanese population suggested that men living without a spouse faced an elevated risk of suicide death (Poudel-Tandukar *et al.*, 2011). In the analysis of middle-aged individuals from the UK Biobank, even after adjusting for loneliness, the increase in suicide rates due to living alone was approximately twice as high for men (Shaw *et al.*, 2021). Furthermore, analysis of American Community Survey data showed an increased hazard of suicide death among individuals living alone (Olfson *et al.*, 2022). These findings underscore the importance of targeted interventions and support for individuals living alone.

Despite the aforementioned findings, two limitations merit attention in this area of research. First, past research only assessed living alone at a given point in time as a time-fixed exposure, controlling for time-fixed confounders. While living arrangements may change over time and be considered as a time-varying variable (Brown *et al.*, 2002; U.S. Census Bureau, 2020), no study has examined how sustained living arrangements and the change in living arrangements impact suicide death. Traditional regression analyses, such as the Cox model, are inadequate for handling time-varying variables, which may introduce bias due to exposure-confounder feedback (Hernán and Robins, 2020). To overcome this limitation, the application of g-methods, such as inverse probability weighting, is necessary (Hernán and Robins, 2020). Second, previous survival analysis studies examining suicide death have only reported a single hazard ratio (HR) averaged over time based on the proportional hazard assumption. Nonetheless, the actual HR may change during the follow-up period, and relying solely on a single HR may potentially lead to misleading interpretations (Hernán, 2010; Prentice *et al.*, 2005). To avoid such interpretations, it is imperative to evaluate the survival and cumulative incidence throughout follow-up periods (Hernán, 2010; Murray *et al.*, 2021).

Given the multifaceted nature of suicide (Shaw, 2022), identifying associated factors is crucial to developing effective prevention strategies. Understanding the association between living alone and suicide death may help identify individuals at a higher risk and enable targeted prevention efforts (Eaton, 2012). By addressing the limitations of past research, we aimed to evaluate the association of time-varying living arrangements with suicide death and examine how this association changed over time. We used multi-point data from the Japan Public Health Center (JPHC)-based Prospective Study, accounting for time-fixed and time-varying confounders in the analyses. To contextualize the association, we also studied non-suicide death and all-cause mortality. Furthermore, given the heterogeneity in suicide rates (World Health Organization, 2021b), we conducted age-stratified and gender-stratified analyses as an exploratory aim.

## Methods

### Study population

We analysed data from the JPHC Study in Japan. The study design has been extensively detailed in a prior study (Tsugane and Sawada, 2014). Participants who completed the questionnaire were considered to have given their consent to participate in the study. This study was approved by the Institutional Review Board of the National Cancer Center and the National Center of Neurology and Psychiatry. Our general population sample comprised individuals from local municipalities in 11 public health centre areas with 2 separate cohorts. Cohort I consisted of individuals aged 40–59 years, while Cohort II consisted of individuals aged 40–69 years. We defined wave 1 as the period of data collection from 1995 to 1999, with the specific timing dependent on each public health centre. Wave 2 was defined as the period 5 years after wave 1, i.e., from 2000 to 2004.

### Cause of death

Our primary outcome was suicide death, while non-suicide death and all-cause mortality were also assessed. All outcomes were evaluated up to 14 years after wave 2. With the permission of the Ministry of Health, Labour and Welfare, we obtained information on the causes of death via death certificates (Sawada *et al.*, 2022). The cause of death was defined according to the International Statistical Classification of Diseases and Related Health Problems, tenth revision (ICD-10) (World Health Organization, 1990). Suicide death was defined using ICD-10 codes X60–X84. Non-suicide death was defined as any death registered under other ICD-10 codes.

### Living arrangements

At waves 1 and 2, we asked participants about their living arrangements by posing the question, ‘Are you currently living with someone (spouse, child(ren), parent(s), others, alone)?’ Here, ‘others’ included both non-family members and additional family members like siblings, grandparents, uncles, aunts, cousins and in-laws.

### Covariates

We evaluated covariates at wave 1 that might serve as potential confounders. We selected covariates per the disjunctive cause criterion, controlling for each potential cause of the exposure, the outcome or both while excluding instrumental variables and including covariates that act as proxies for unmeasured variables that are common causes of both the exposure and the outcome (VanderWeele, 2019). We included the following variables that would fulfil the disjunctive cause criterion: age (continuous), gender (dichotomous, female or male), body mass index (continuous) (Amiri and Behnezhad, 2018), smoking status (dichotomous, no or yes) (Harrison *et al.*, 2020), alcohol consumption (dichotomous, <once a week or ≥once a week) (Isaacs *et al.*, 2022), physical activity (dichotomous, <once a week or ≥once a week) (Vancampfort *et al.*, 2018), employment status (dichotomous, employed or homemaker or unemployed) (Schneider *et al.*, 2011), sleep duration (dichotomous, >6 hours or ≤6 hours) (Dolsen *et al.*, 2021), history of cancer (dichotomous, no or yes) (Zaorsky *et al.*, 2019), history of cerebrovascular or cardiovascular disease (dichotomous, no or yes) (Chan *et al.*, 2007; Wu et al., 2018) and consumption of vegetables (continuous) (Nanri *et al.*, 2013), fruits (continuous) (Narita *et al.*, 2022), fish (continuous) (Nanri *et al.*, 2013) and meat (continuous) (Molendijk *et al.*, 2018), as well as region (categorical, Ninohe, Yokote, Saku, Chubu, Katsushika, Mito, Nagaoka, Chuo-higashi, Kamigoto, Miyako and Suita) (Fedina *et al.*, 2021; Narita *et al.*, 2020a; Yoshioka *et al.*, 2021). Employment status was included as a surrogate for socio-economic status (Bartley and Owen, 1996), for which data were not available. Food consumption was determined by multiplying the frequency of consumption by the relative portion size and energy-adjusted using the residual method (Willett *et al.*, 1997).

Moreover, we controlled for the following factors at wave 2 as time-varying confounders, which may be affected by living arrangements at wave 1 and also confound the association of living arrangements at wave 2 with suicide death, non-suicide death and all-cause mortality: smoking status, employment status, sleep duration, history of cancer, history of cerebrovascular and cardiovascular disease and consumption of vegetables, fruits, fish and meat.

### Statistical analyses

**Figure 1.**
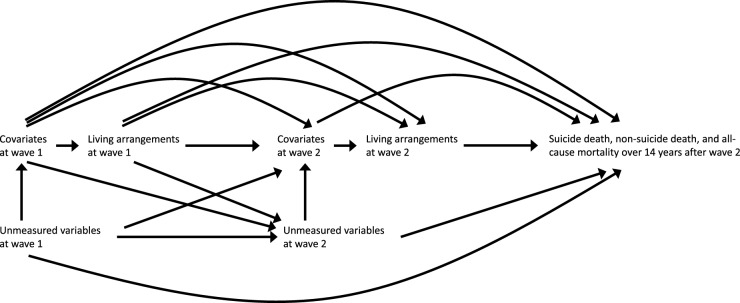
A causal directed acyclic graph assumed for the main analysis. Wave 1 was defined as the period from 1995 to 1999, depending on the timing of data collection at each public health centre area, while wave 2 was defined as 5 years after wave 1, from 2000 to 2004.

All analyses were conducted using R-4.3.1. We used inverse probability weighted pooled logistic regression models for discrete-time hazards (Murray *et al.*, 2021). Thereby, we evaluated the association of living arrangements at waves 1 and 2 with suicide death, non-suicide death and all-cause mortality during the 14-year follow-up. The directed acyclic graph of the hypothesized associations is shown in Fig. 1. We used random forest imputation (Stekhoven and Bühlmann, 2012) to impute missing data for covariates but not for living arrangements and the number of years followed because they might be caused by the occurrence of events during waves 1 and 2.

Our models controlled for time-fixed and time-varying confounders using inverse probability weighting (Hernán and Robins, 2020). We calculated stabilized weights for living alone at waves 1 and 2 utilizing separate logistic models for exposures at waves 1 (adjusting for covariates at wave 1) and 2 (adjusting for exposure at wave 1 and covariates at waves 1 and 2). Since the number of regions was small in our data, we used fixed effects models to account for regions, which would safeguard against bias for the estimates (McNeish and Kelley, 2019). To address selection bias due to loss to follow-up, we further generated cumulative stabilized weights for censoring, the value of which varied during the follow-up. Each weight was truncated at the 99th percentile (Cole and Hernán, 2008). The final stabilized weights were obtained as the product of these stabilized weights for censoring up to each follow-up year and the stabilized weights for living arrangements. This weighting produced a pseudo-population where covariates are balanced between groups and between those with and without censoring. A variable for year *k* (*k* = 0, 1, …, 14) was created as a continuous variable. The weighted pooled logistic models of the outcome were fit on the exposures and time in years (*k*), to adjust for changing hazards over time. See Supplementary Appendix 1 for details on our models.

To elucidate the performance of the pooled logistic model in predicting a survival function, we compared the Kaplan–Meier curve and the survival curve from unweighted pooled logistic regression. Next, based on the inverse probability weighted pooled logistic models, we predicted counterfactual hazard at each year and calculated survival probability that would have been observed if everyone had a specific pattern of living arrangements, namely: (1) living with someone at both waves 1 and 2, (2) living with someone at wave 1 but alone at wave 2, (3) living alone at wave 1 but with someone at wave 2 and (4) living alone at both waves 1 and 2. This approach allowed us to obtain cumulative incidence, risk differences (RDs) and risk ratios (RRs) throughout the follow-up period. We calculated RDs and RRs at 7 and 14 years after wave 2 (the midpoint and the end of the follow-up period) with pointwise bootstrap confidence intervals (CIs) using percentiles at each time.

Next, we conducted a sensitivity analysis controlling for additional baseline factors, namely prior mental illness, social support and coping. These factors might have been potential confounders (Kleiman and Liu, 2013; Narita *et al.*, 2023, 2020b; Stanley *et al.*, 2021) but were not measured at wave 1. Thus, we employed proxies for these factors. For the proxy of prior mental illness, we controlled for self-reported stress level (categorical, low, medium or high) and life enjoyment (categorical, low, medium or high) at wave 1. For the proxy of social support, we controlled for social support (continuous) 5 years prior to wave 1. Social support was evaluated using self-reported items that measured confidant support, esteem support and social isolation; details are available elsewhere (Ikeda *et al.*, 2013). Since this information was only available in Cohort II, one of the two cohorts constituting our general population sample, the missing data were handled via random forest imputation. For the proxy of coping, we controlled for approach-oriented coping (continuous) and avoidance-oriented coping (continuous) at wave 2, which were evaluated using self-reported items selected from the validated Japanese version of the Stress and Coping Inventory (Fukunishi *et al.*, 1995); details are available elsewhere (Shikimoto *et al.*, 2022). Our sensitivity analysis adjusted for mental illness and social support proxies at baseline in the wave-1 and -2 exposure models, and for coping variables after baseline only in the wave-2 exposure model. As coping variables were controlled through inverse probability weighting, we partially adjusted for potential confounding due to these variables without conditioning on mediators.

We examined the robustness of the estimates to potential unmeasured confounding by calculating E-values (VanderWeele and Ding, 2017). This involved determining the minimum strength of the association that an unmeasured confounder would require to have above and beyond the measured covariates to explain away the estimates. In other words, E-values quantify the degree of influence an unmeasured confounder, not included in our study, would need to have to nullify our findings, thereby offering a measure of the robustness of the results.

Finally, we evaluated age-stratified and gender-stratified associations. Because of the limited number of age-specific and gender-specific suicide incidences, we chose parsimonious models after iteratively fitting pooled logistic regression models with inverse probability weighting for each stratum. The threshold for age was set at 60, as this was the standard retirement age in Japan.

## Results

We identified 103,880 participants. Table 1 summarizes the demographic features of the study participants at wave 1, categorized by living arrangements at waves 1 and 2. Data for living arrangements were missing for 16,590 participants (16.0%). The mean age (standard deviation) was 51.7 (7.90). The number of males was 55,352 (53.3%), while females numbered 48,528 (46.7%). Participants who lived alone at both waves 1 and 2 were characterized by older age, a higher likelihood of being female, higher engagement in physical exercise and a higher likelihood of being unemployed or a homemaker compared with those who lived with someone at both time points. We did not observe substantial differences in other characteristics among the groups. Note that the details of missing covariates are also shown in Table 1. Of the identified participants, 86,749 with data for living arrangements at both waves and data for the number of years followed were used for the analysis, while missing data for covariates were handled using random forest imputation. The flow diagram of the study population selection is summarized in Supplementary Fig. S1.

**Table 1.** Demographic features of study participants at wave 1 by living arrangements at waves 1 and 2

		Living arrangements				
Covariates	Overall (*N* = 103,880)	Living with someone in both waves 1 and 2 (*N* = 80,666)	Living with someone at wave 1 but alone at wave 2 (*N* = 2531)	Living alone at wave 1 but with someone at wave 2 (*N* = 1033)	Living alone in both waves 1 and 2 (*N* = 3060)	Missing (*N* = 16,590)
Age (years), mean (SD)	51.7 (7.90)	51.5 (7.70)	54.2 (8.38)	53.4 (8.53)	56.1 (8.19)	51.7 (8.34)
Gender, *n* (%)						
Female	55,352 (53.3)	42,780 (53.0)	1780 (70.3)	661 (64.0)	2253 (73.6)	7878 (47.5)
Male	48,528 (46.7)	37,886 (47.0)	751 (29.7)	372 (36.0)	807 (26.4)	8712 (52.5)
Body mass index, mean (SD)	23.5 (3.09)	23.6 (3.04)	23.4 (3.21)	23.6 (3.07)	23.4 (3.27)	23.3 (3.27)
Missing, *n* (%)	3003 (2.9)	1993 (2.5)	120 (4.7)	49 (4.7)	137 (4.5)	704 (4.2)
Smoking status, *n* (%)						
No	73,107 (70.4)	57,769 (71.6)	1816 (71.8)	707 (68.4)	2192 (71.6)	10,623 (64.0)
Yes	24,163 (23.3)	18,157 (22.5)	432 (17.1)	237 (22.9)	537 (17.5)	4800 (28.9)
Missing	6610 (6.4)	4740 (5.9)	283 (11.2)	89 (8.6)	331 (10.8)	1167 (7.0)
Alcohol consumption, *n* (%)						
Less than once a week	62,325 (60.0)	48,329 (59.9)	1705 (67.4)	644 (62.3)	2175 (71.1)	9472 (57.1)
Once a week or more	38,965 (37.5)	30,539 (37.9)	673 (26.6)	354 (34.3)	765 (25.0)	6634 (40.0)
Missing	2590 (2.5)	1798 (2.2)	153 (6.0)	35 (3.4)	120 (3.9)	484 (2.9)
Physical activity, *n* (%)						
Less than once a week	76,600 (73.7)	59,921 (74.3)	1738 (68.7)	763 (73.9)	2110 (69.0)	12,068 (72.7)
Once a week or more	21,583 (20.8)	16,690 (20.7)	508 (20.1)	230 (22.3)	814 (26.6)	3341 (20.1)
Missing	5697 (5.5)	4055 (5.0)	285 (11.3)	40 (3.9)	136 (4.4)	1181 (7.1)
Employment status, *n* (%)						
Employed	71,826 (69.1)	56,296 (69.8)	1658 (65.5)	726 (70.3)	1840 (60.1)	11,306 (68.1)
Homemaker or unemployed	32,054 (30.9)	24,370 (30.2)	873 (34.5)	307 (29.7)	1220 (39.9)	5284 (31.9)
Sleep duration, *n* (%)						
Longer than 6 hours	72,201 (69.5)	57,257 (71.0)	1622 (64.1)	674 (65.2)	2017 (65.9)	10,631 (64.1)
6 hours or shorter	28,636 (27.6)	21,327 (26.4)	745 (29.4)	339 (32.8)	999 (32.6)	5226 (31.5)
Missing	3043 (2.9)	2082 (2.6)	164 (6.5)	20 (1.9)	44 (1.4)	733 (4.4)
History of cancer, *n* (%)						
No	102,365 (98.5)	79,663 (98.8)	2493 (98.5)	1022 (98.9)	3023 (98.8)	16,164 (97.4)
Yes	1515 (1.5)	1003 (1.2)	38 (1.5)	11 (1.1)	37 (1.2)	426 (2.6)
History of cerebrovascular or cardiovascular disease, *n* (%)						
No	100,838 (97.1)	78,466 (97.3)	2451 (96.8)	1004 (97.2)	2980 (97.4)	15,937 (96.1)
Yes	3042 (2.9)	2200 (2.7)	80 (3.2)	29 (2.8)	80 (2.6)	653 (3.9)
Vegetable consumption (g/d), mean (SD)	207 (134)	210 (133)	213 (148)	198 (134)	211 (150)	193 (134)
Missing, *n* (%)	1089 (1.0)	696 (0.9)	78 (3.1)	7 (0.7)	10 (0.3)	298 (1.8)
Fruit consumption (g/d), mean (SD)	205 (170)	207 (168)	227 (198)	213 (179)	225 (184)	186 (171)
Missing, *n* (%)	1089 (1.0)	696 (0.9)	78 (3.1)	7 (0.7)	10 (0.3)	298 (1.8)
Fish consumption (g/d), mean (SD)	85.1 (52.8)	85.8 (51.5)	87.1 (59.9)	81.6 (55.4)	81.1 (54.0)	82.5 (57.0)
Missing, *n* (%)	1089 (1.0)	696 (0.9)	78 (3.1)	7 (0.7)	10 (0.3)	298 (1.8)
Meat consumption (g/d), mean (SD)	57.0 (39.6)	56.8 (38.7)	55.1 (40.1)	56.1 (45.0)	51.2 (39.6)	59.8 (42.9)
Missing, *n* (%)	1089 (1.0)	696 (0.9)	78 (3.1)	7 (0.7)	10 (0.3)	298 (1.8)

Abbreviation: SD, standard deviation. Wave 1 was defined as 1995–1999, depending on the timing of the survey conducted at each public health centre area, and wave 2 was defined as 5 years after wave 1, i.e., 2000–2004.

### Cumulative incidence over time

**Figure 2.**
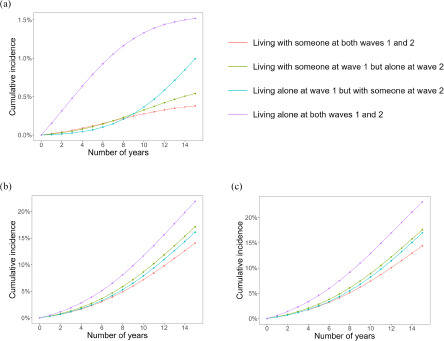
The cumulative incidence of suicide death (a), non-suicide death (b) and all-cause mortality (c) estimated using the inverse probability weighted pooled logistic regression, incorporating covariates measured at waves 1 (1995–1999) and 2 (2000–2004) into the weights for time-varying exposures (waves 1 and 2) and censoring during the follow-up period (up to 14 years after wave 2).

During the follow-up period, a total of 17,332 participants died, out of which 306 died by suicide, while 17,026 had non-suicide deaths. The mean (standard deviation) duration of the follow-up period was 12.7 (3.09) years. Supplementary Figure S2 shows the comparison of the Kaplan–Meier curve and the survival curve from unweighted pooled logistic regression. For all types of mortality, these curves appeared to have similar trajectories, indicating the good performance of our pooled logistic model. Figure 2 displays the cumulative incidence curves based on the pooled logistic model. It was observed that individuals living alone at both waves 1 and 2 consistently had a higher incidence rate compared with those who lived with someone over time. On the other hand, the findings were not as substantial for those living alone at either wave 1 or 2, especially in the first half of the follow-up period. While non-suicide death and all-cause mortality displayed comparable trajectories, the difference in cumulative incidence appeared to be particularly remarkable for suicide death at the end of the follow-up period.

### Risk at the midpoint and the end of the follow-up period

Table 2 summarizes RDs and RRs at the midpoint and the end of the follow-up period from the inverse probability weighted cumulative incidence curves. Individuals who consistently lived alone exhibited a higher risk of suicide death, with an observed four-fold increase in risk at the end of the follow-up period (RD: 1.1%, 95% CI: 0.3–2.5%; RR: 4.00, 95% CI: 1.83–7.41). While findings were comparable at the midpoint of the follow-up period, the findings at this point should be interpreted with caution due to the limited number of suicide deaths, resulting in wide CIs. The risk in those who consistently lived alone was increased for non-suicide death (RD: 7.8%, 95% CI: 5.2–10.5%; RR: 1.56, 95% CI: 1.38–1.74) and all-cause mortality (RD: 8.7%, 95% CI: 6.2–11.3%; RR: 1.60, 95% CI: 1.42–1.79) at the follow-up period, with narrower CIs due to a greater number of cases. For suicide deaths, the increased risk observed in those who started to live with someone and those who transitioned to living alone was less impactful and did not reach statistical significance. For non-suicide death and all-cause mortality, those who transitioned to living alone showed a small but increased risk (non-suicide death: RD: 3.1%, 95% CI: 1.7–4.8%; RR: 1.22, 95% CI: 1.12–1.34; all-cause mortality: RD: 3.2%, 95% CI: 1.7–4.9%; RR: 1.22, 95% CI: 1.12–1.34), whereas those who started to live with someone showed non-significant results.

**Table 2.** Association of time-varying living arrangements at waves 1 and 2 with suicide death, non-suicide death and all-cause mortality

			Suicide death			Non-suicide death			All-cause mortality		
Living arrangements at wave 1	Living arrangements at wave 2	Person-years	Number of deaths	RD (95% bootstrap CI), %	RR (95% bootstrap CI)	Number of deaths	RD (95% bootstrap CI), %	RR (95% bootstrap CI)	Number of deaths	RD (95% bootstrap CI), %	RR (95% bootstrap CI)
			7 years after wave 2 (the midpoint of the follow-up period)								
Living with someone	Living with someone	620,275	163	0.0	1.00	5214	0.0	1.00	5377	0.0	1.00
Living with someone	Living alone	19,190	6	0.1 (−0.2, 0.2)	1.06 (0.33, 1.95)	198	1.0 (0.1, 1.9)	1.20 (1.02, 1.39)	204	1.0 (0.2, 2.0)	1.19 (1.03, 1.40)
Living alone	Living with someone	7605	2	0.0 (−0.2, 0.6)	0.95 (1e−6, 3.77)	91	0.4 (−1.4, 2.8)	1.09 (0.72, 1.56)	93	0.4 (−1.3, 2.7)	1.08 (0.74, 1.54)
Living alone	Living alone	23,193	9	0.9 (0.2, 2.1)	5.39 (1.73, 10.51)	279	3.2 (1.7, 5.0)	1.65 (1.35, 2.04)	288	4.1 (2.5, 5.9)	1.80 (1.50, 2.15)
			14 years after wave 2 (the end of the follow-up period)								
Living with someone	Living with someone	1,100,640	276	0.0	1.00	15,365	0.0	1.00	15,641	0.0	1.00
Living with someone	Living alone	33,437	11	0.2 (0.1, 0.5)	1.43 (0.65, 2.32)	563	3.1 (1.7, 4.8)	1.22 (1.12, 1.34)	574	3.2 (1.7, 4.9)	1.22 (1.12, 1.34)
Living alone	Living with someone	13,196	5	0.6 (−0.3, 1.8)	2.62 (0.16, 6.00)	246	2.0 (−1.5, 6.0)	1.15 (0.90, 1.42)	251	2.6 (−0.9, 6.3)	1.18 (0.93, 1.44)
Living alone	Living alone	39,780	14	1.1 (0.3, 2.5)	4.00 (1.83, 7.41)	852	7.8 (5.2, 10.5)	1.56 (1.38, 1.74)	866	8.7 (6.2, 11.3)	1.60 (1.42, 1.79)

Abbreviations: RD, risk difference; RR, risk ratio; CI, confidence interval; NA, not applicable.

Wave 1 was defined as the period from 1995 to 1999, depending on the timing of data collection at each public health centre area, while wave 2 was defined as 5 years after wave 1, from 2000 to 2004.

The RD and RR were calculated based on cumulative incidence functions estimated by the inverse probability of exposure and censoring weighted pooled logistic regression accounting for covariates measured at waves 1 and 2 in the weights.

Age, gender, body mass index, smoking status, alcohol consumption, physical activity, employment status, sleep duration, history of cancer, history of cerebrovascular or cardiovascular disease, vegetable consumption, fruit consumption, fish consumption, meat consumption and region at wave 1 were controlled for. Also, smoking status, employment status, sleep duration, history of cancer, history of cerebrovascular or cardiovascular disease, vegetable consumption, fruit consumption, fish consumption and meat consumption at wave 2 were controlled for because these factors may be affected by living arrangements at wave 1 and also confound the association of living arrangements at wave 2 with suicide death, non-suicide death and all-cause mortality.

### Sensitivity analysis

Next, we conducted a sensitivity analysis to control for proxies of prior mental illness, social support and coping. Supplementary Table S1 summarizes the results of this analysis. In short, the findings remained robust, with the RD and RR at both the midpoint and the end of the follow-up period showing no substantial changes when these potential confounders were incorporated into the models. The findings were consistent across all types of mortality.

### Robustness to unmeasured confounders

The calculated E-values showed that some observed associations between living arrangements and suicide death, non-suicide death and all-cause mortality were reasonably robust to unmeasured confounders (see Table 3). For example, for the association of living alone at both waves 1 and 2 with suicide death at the end of the follow-up period, an unmeasured confounder would need to be associated with both of them above and beyond the adjusted covariates by an RR of 7.46 to fully explain away the observed association and 3.06 to shift the CI to include the null value.

**Table 3.** Robustness to unmeasured confounding of the association of living arrangements at waves 1 and 2 with suicide death, non-suicide death and all-cause mortality

Living arrangements at wave 1	Living arrangements at wave 2	E-value	Suicide death	Non-suicide death	All-cause mortality
		7 years after wave 2 (the midpoint of the follow-up period)			
Living with someone	Living alone	Point estimate	1.31	1.69	1.67
		Limit of CI	1.00	1.16	1.21
Living alone	Living with someone	Point estimate	1.29	1.40	1.37
		Limit of CI	1.00	1.00	1.00
Living alone	Living alone	Point estimate	10.25	2.69	3.00
		Limit of CI	2.85	2.04	2.37
		14 years after wave 2 (the end of the follow-up period)			
Living with someone	Living alone	Point estimate	2.21	1.74	1.74
		Limit of CI	1.00	1.49	1.49
Living alone	Living with someone	Point estimate	4.68	1.57	1.64
		Limit of CI	1.00	1.00	1.00
Living alone	Living alone	Point estimate	7.46	2.49	2.58
		Limit of CI	3.06	2.10	2.19

Abbreviation: CI, confidence interval.

E-values for point estimates are the minimum strength of association on the RR that unmeasured confounding would need to have above and beyond the adjusted covariates to explain away the estimates. E-values for the limit of CI are the minimum strength of association on the RR that unmeasured confounding would need to have above and beyond the adjusted covariates to shift the 95% CI and include the null value.

### Age-stratified and gender-stratified associations

The age-stratified associations (60 or older vs. 59 or younger) are presented in Supplementary Tables S2 and S3. The results for participants aged 60 or older were inconsistent, likely due to the limited number of cases, whereas the findings for individuals aged 59 or younger were similar to those of the main analyses. Supplementary Tables S4 and S5 present the gender-stratified associations. Overall, the results appeared inconsistent. The limited number of suicide deaths warrants caution in the interpretation of these findings.

## Discussion

In this cohort study comprising a general population sample, the inverse probability weighted pooled logistic regression and the cumulative incidence curve allowed us to evaluate the impact of time-varying living arrangements over time, controlling for time-varying confounders. The performance of our pooled logistic model was shown by comparing the survival curve from the unweighted model with the Kaplan–Meier curve. While the cumulative incidence curve showed the dynamics of the impact of living arrangements, those who lived alone at both waves 1 and 2 consistently showed a higher cumulative incidence rate than those who persistently lived with someone throughout the study period. The computation of risk at the midpoint and the end of the follow-up period showed that persistently living alone at both waves 1 and 2 was associated with an increased risk of suicide death compared to those who persistently lived with someone (0.9–1.1% absolute risk increase and more than a four-fold relative risk increase). On the other hand, this association was weaker and non-significant for those who changed living arrangements (up to 0.6% absolute risk increase and up to a 2.6-fold relative risk increase). Those who persistently lived alone showed an increased risk of non-suicide death and all-cause mortality (3.2–8.7% absolute risk increase and more than a 1.5-fold relative risk increase), suggesting that living alone is a pervasive risk factor for various causes of death. The robustness of the findings was supported via the sensitivity analysis controlling for the proxies of prior mental illness, social support and coping. The robustness was further verified by calculating E-values. While previous research has shown an association between living arrangements and suicide death (Olfson *et al.*, 2022; Poudel-Tandukar *et al.*, 2011; Shaw *et al.*, 2021), this study is the first to examine living arrangements as a time-varying variable, controlling for time-fixed and time-varying confounders, and to evaluate the changing association over time.

In this study, 3,507 participants changed their living arrangements from wave 1 to wave 2. Such participants did not show an increased risk of suicide death in the first half of the follow-up period. Even at the end of the follow-up period, the impact was not as substantial as for those who persistently lived alone. Thus, living arrangements may change, and this variability indeed impacts the risk of suicide death. A similar discussion applies to non-suicide death and all-cause mortality. These findings hold significant importance, particularly considering that living alone represents an intervenable factor that can be effectively addressed through approaches in public health and social work (Nestadt, 2022).

Previous research examining suicide death using survival analysis has reported a single average HR over the follow-up period. However, the causal interpretation of this single HR may potentially be misleading due to its variation over time (Hernán, 2010; Prentice *et al.*, 2005). Consequently, it is crucial to account for time-dependent HRs. Nonetheless, presenting only a single time-dependent HR can also be misleading due to built-in selection bias, which arises from the selection of participants at higher risk of events earlier in the study (Hernán, 2010; Prentice *et al.*, 2005). To address these issues, we employed a discrete-time hazard model (Murray *et al.*, 2021), showing the cumulative incidence over time and calculating RD and RR at multiple time points. Our cumulative incidence curve provides insights into the dynamics of the association between living arrangements and suicide death throughout the follow-up period.

The heterogeneous suicide rates across ages (World Health Organization, 2021b) compelled us to explore the age-stratified associations. While the results for participants aged 59 or younger were similar to those of the main analyses, the limited number of cases led to inconsistent results in those aged 60 or older. In addition, the same issue prevented the inclusion of the product term of the year and living arrangements variables, hindering the analysis of time-dependent HR. Taken together, these findings should be regarded as exploratory results, and caution should be exercised when interpreting and comparing the results. The same discussion applies to the gender-stratified associations. Past research has suggested an association between living arrangements and suicide death, particularly in men (Poudel-Tandukar *et al.*, 2011; Shaw *et al.*, 2021). While effect heterogeneity across gender is possible, the limited number of cases again impeded obtaining reliable results. Future studies should include an adequate number of cases to incorporate the product term of the year and living arrangements variables, accounting for time-dependent HR.

Two mechanisms may be proposed to explain the association between living alone and suicide death. The interpersonal theory of suicide suggests that the risk of suicide is influenced by various social factors, including thwarted belongingness, perceived burdensomeness and hopelessness (Van Orden *et al.*, 2010). Living alone may increase the likelihood of social isolation and loneliness, which can correlate with a sense of thwarted belongingness. Indeed, social isolation and loneliness are associated with detrimental mental health and suicide-related outcomes (Narita *et al.*, 2021, 2020c; Solmi *et al.*, 2020; Stickley and Koyanagi, 2016). The integrated motivational–volitional theory distinguishes between the premotivational, motivational and volitional phases in the progression of suicide development (O’Connor and Kirtley, 2018). Among these phases, the volitional phase is characterized by the transition of thoughts about suicidal intent into actual actions (O’Connor and Kirtley, 2018). We did not conduct a mediation analysis in this study because mediation using a discrete-time hazard model may require additional development. However, employing such an analysis in a different framework could enhance scientific understanding.

The present study has implications for public health and social work, as well as for the fields of psychiatry and psychology. Our study showed that persistently living alone had a substantial impact on suicide death, emphasizing its importance in the context of prevention. The identification of individuals at higher risk of suicide enables targeted prevention efforts (Eaton, 2012). Living alone is a prevalent condition, exceeding 30% in Japan (Ministry of Health, Labour and Welfare, 2022). This widespread distribution would make targeted prevention impactful. Living alone is an intervenable factor (Nestadt, 2022), and the present study indeed showed that transitions in living arrangements can substantially change the risk of suicide death, providing warranted practical evidence (Olfson *et al.*, 2022). While addressing the challenges of living alone at the national level is beyond the scope of this paper, social programmes that provide a sense of community and improved access to mental health services might be beneficial (Kleiman and Liu, 2013; Tadmon and Bearman, 2023). For clinicians, our findings highlight the need to consider patients’ living situations as a factor that may contribute to suicide risk. Psychologists, too, can incorporate these insights into therapeutic approaches, potentially targeting feelings of isolation or developing strategies to foster social connection for individuals living alone. Further, our findings may help guide future studies investigating social factors that can be intervened upon to mitigate the impact of living alone, including causal mediation analysis.

### Limitations

Our study has several limitations. First, measurement bias is conceivable not only in self-reported living arrangements but also in suicide deaths obtained from death certificates (Shaw *et al.*, 2022). Second, certain potential confounders were not accounted for due to the lack of available information, e.g., childhood maltreatment (Stickley *et al.*, 2021, 2020). For socio-economic status (Rehkopf and Buka, 2006), we used employment status as a surrogate (Bartley and Owen, 1996). For prior mental illness, social support, and coping (Kleiman and Liu, 2013; Narita *et al.*, 2023, 2020b; Stanley *et al.*, 2021), we showed the robustness of the findings via a sensitivity analysis incorporating the proxies of these potential confounders at wave 1. Also, E-values suggested the robustness of the findings against unmeasured potential confounders. Taken together, although the influence of unmeasured confounders is conceivable, it is unlikely to substantially change the conclusion. Third, the limited number of cases hindered our ability to provide comprehensive information on certain results. This limitation was particularly relevant when analysing suicide death in those who changed living arrangements, which resulted in small statistical power and wide CIs. Similarly, the age-stratified and gender-stratified analyses provided inconsistent results, likely due to this limitation. Also, we were unable to construct a model that examines how the impact may vary depending on other factors, including social support and coping strategies (Kleiman and Liu, 2013; Narita *et al.*, 2023; Stanley *et al.*, 2021). Fourth, we maintained the same 5-year interval for evaluating living arrangements and the 14-year follow-up for every public health centre area, but the initiation of data collection varied between areas. While this variability was controlled for in the model, it did not address the variations in suicide trends towards the end of the follow-up period (i.e., 2014–2018). In Japan, the number of suicides in 2014 was 25,427 with a rate of 20.0 per 100,000 individuals; by 2018, they dropped to 20,840 and 16.5 per 100,000 individuals, respectively (National Police Agency, 2023), which might have influenced the results. Fifth, we handled missing covariate data using random forest imputation but chose not to impute missing exposure data due to potential events occurring between waves 1 and 2. While the demographics did not show substantial differences between the analysed and missing data, the selection of participants might have somewhat influenced the results. Sixth, we evaluated living arrangements as time-varying variables at waves 1 and 2. However, it is possible that some participants could have changed living arrangements multiple times between these waves, which was not fully captured. Finally, this study is population-based but only includes individuals aged 40–69 years at baseline, limiting the generalizability of the results to other age groups.

## Conclusions

Individuals who persistently live alone have an increased risk of suicide death, non-suicide death and all-cause mortality, whereas this impact is weaker for those who change their living arrangements.

## Supporting information

Narita et al. supplementary material Narita et al. supplementary material

## Data Availability

For information on how to apply to gain access to JPHC data, follow the instructions at https://epi.ncc.go.jp/en/jphc/805/8155.html.

## Supplementary material

The supplementary material for this article can be found at https://doi.org/10.1017/S2045796024000325.

## References

[ref1] Amiri S and Behnezhad S (2018) Body mass index and risk of suicide: A systematic review and meta-analysis. *Journal of Affective Disorders* 238, 615–625. 29957479 10.1016/j.jad.2018.05.028

[ref2] Bartley M and Owen C (1996) Relation between socioeconomic status, employment, and health during economic change, 1973-93. *BMJ* 313, 445–449. 8776309 10.1136/bmj.313.7055.445 PMC2351866

[ref3] Brown JW, Liang J, Krause N, Akiyama H, Sugisawa H and Fukaya T (2002) Transitions in living arrangements among elders in Japan: Does health make a difference? *The Journals of Gerontology Series B, Psychological Sciences and Social Sciences* 57, S209–20. 12084791 10.1093/geronb/57.4.s209

[ref4] Chan SS, Lyness JM and Conwell Y (2007) Do cerebrovascular risk factors confer risk for suicide in later life? A case-control study. *The American Journal of Geriatric Psychiatry: Official Journal of the American Association for Geriatric Psychiatry* 15, 541–544. 17545453 10.1097/JGP.0b013e31803c5523

[ref5] Cole SR and Hernán MA (2008) Constructing inverse probability weights for marginal structural models. *American Journal of Epidemiology* 168, 656–664. 18682488 10.1093/aje/kwn164 PMC2732954

[ref6] Dolsen EA, Prather AA, Lamers F and Penninx BWJH (2021) Suicidal ideation and suicide attempts: Associations with sleep duration, insomnia, and inflammation. *Psychological Medicine* 51, 2094–2103. 32321599 10.1017/S0033291720000860

[ref7] Eaton WW (ed.) (2012) *Public Mental Health*. New York: Oxford University Press.

[ref8] Fedina L, Mushonga DR, Bessaha ML, Jun H-J, Narita Z and DeVylder J (2021) Moderating effects of perceived neighborhood factors on intimate partner violence, psychological distress, and suicide risk. *Journal of Interpersonal Violence* 36, 10546–10563. 31686578 10.1177/0886260519884687

[ref9] Fukunishi I, Nakagawa T, Nakagawa H, Sone Y, Kaji N, Hosaka T and Rahe RH (1995) Validity and reliability of the Japanese version of the Stress and Coping Inventory. *Psychiatry and Clinical Neurosciences.* 49, 195–199. 9179937 10.1111/j.1440-1819.1995.tb01884.x

[ref10] Harrison R, Munafò MR, Davey Smith G and Wootton RE (2020) Examining the effect of smoking on suicidal ideation and attempts: Triangulation of epidemiological approaches. *The British Journal of Psychiatry: The Journal of Mental Science* 217, 701–707. 32290872 10.1192/bjp.2020.68 PMC7705667

[ref11] Hernán MA (2010) The hazards of hazard ratios. *Epidemiology* 21, 13–15. 20010207 10.1097/EDE.0b013e3181c1ea43 PMC3653612

[ref12] Hernán M, and Robins J (2020) *Causal Inference: What If*, Boca R (ed.). FL: Chapman & Hall/CRC, 257–275.

[ref13] Ikeda A, Kawachi I, Iso H, Iwasaki M, Inoue M and Tsugane S (2013) Social support and cancer incidence and mortality: The JPHC study cohort II. *Cancer Causes and Control* 24, 847–860. 23549959 10.1007/s10552-013-0147-7

[ref14] Isaacs JY, Smith MM, Sherry SB, Seno M, Moore ML and Stewart SH (2022) Alcohol use and death by suicide: A meta-analysis of 33 studies. *Suicide and Life-Threatening Behavior* 52, 600–614. 35181905 10.1111/sltb.12846

[ref15] Kleiman EM and Liu RT (2013) Social support as a protective factor in suicide: Findings from two nationally representative samples. *Journal of Affective Disorders* 150, 540–545. 23466401 10.1016/j.jad.2013.01.033 PMC3683363

[ref16] McNeish D and Kelley K (2019) Fixed effects models versus mixed effects models for clustered data: Reviewing the approaches, disentangling the differences, and making recommendations. *Psychological Methods* 24, 20–35. 29863377 10.1037/met0000182

[ref17] Ministry of Health, Labour and Welfare (2022) Comprehensive survey of living conditions. https://www.mhlw.go.jp/toukei/saikin/hw/k-tyosa/k-tyosa22/dl/02.pdf (Japanese) (accessed 15 May 2024).

[ref18] Molendijk M, Molero P, Ortuño Sánchez-Pedreño F, Van der Does W and Angel Martínez-González M (2018) Diet quality and depression risk: A systematic review and dose-response meta-analysis of prospective studies. *Journal of Affective Disorders* 226, 346–354. 29031185 10.1016/j.jad.2017.09.022

[ref19] Murray EJ, Caniglia EC and Petito LC (2021) Causal survival analysis: A guide to estimating intention-to-treat and per-protocol effects from randomized clinical trials with non-adherence. *Research Methods in Medicine & Health Sciences* 2, 39–49.

[ref20] Nanri A, Mizoue T, Poudel-Tandukar K, Noda M, Kato M, Kurotani K, Goto A, Oba S, Inoue M, Tsugane S and Japan Public Health Center-based Prospective Study Group (2013) Dietary patterns and suicide in Japanese adults: The Japan Public Health Center-based Prospective Study. *The British Journal of Psychiatry: The Journal of Mental Science* 203, 422–427. 24115342 10.1192/bjp.bp.112.114793

[ref21] Narita Z, Banawa R, Zhou S, DeVylder J, Koyanagi A and Oh H (2021) Loneliness and psychotic experiences among US university students: Findings from the Healthy Minds Study 2020. *Psychiatry Research* 308, 114362. 10.1016/j.psychres.2021.114362 34974410

[ref22] Narita Z, Devylder J, Bessaha M and Fedina L (2023) Associations of self-isolation, social support and coping strategies with depression and suicidal ideation in U.S. young adults during the COVID-19 pandemic. *International Journal of Mental Health Nursing* 32, 929–937. 36939066 10.1111/inm.13138

[ref23] Narita Z, Knowles K, Fedina L, Oh H, Stickley A, Kelleher I and DeVylder J (2020a) Neighborhood change and psychotic experiences in a general population sample. *Schizophrenia Research* 216, 316–321. 31791815 10.1016/j.schres.2019.11.036

[ref24] Narita Z, Nozaki S, Shikimoto R, Hori H, Kim Y, Mimura M, Tsugane S and Sawada N (2022) Association between vegetable, fruit, and flavonoid-rich fruit consumption in midlife and major depressive disorder in later life: The JPHC Saku Mental Health Study. *Translational Psychiatry* 12, 412. 10.1038/s41398-022-02166-8 PMC9512814 36163244

[ref25] Narita Z, Stickley A and DeVylder J (2020c) Loneliness and psychotic experiences in a general population sample. *Schizophrenia Research* 218, 146–150. 32014362 10.1016/j.schres.2020.01.018

[ref26] Narita Z, Wilcox HC and DeVylder J (2020b) Psychotic experiences and suicidal outcomes in a general population sample. *Schizophrenia Research* 215, 223–228. 31668492 10.1016/j.schres.2019.10.024

[ref27] National Police Agency (2023) The number of suicide deaths. https://www.npa.go.jp/publications/statistics/safetylife/jisatsu.html (Japanese) (accessed 15 May 2024).

[ref28] Nestadt PS (2022) Suicide and the solitary life: Differential risks of living alone across sociodemographic groups. *American Journal of Public Health* 112, 1702–1704. 36383943 10.2105/AJPH.2022.307136 PMC9670216

[ref29] O’Connor RC and Kirtley OJ (2018) The integrated motivational–volitional model of suicidal behaviour. *Philosophical Transactions of the Royal Society of London Series B, Biological Sciences* 373, 20170268. 10.1098/rstb.2017.0268 PMC6053985 30012735

[ref30] Olfson M, Cosgrove CM, Altekruse SF, Wall MM and Blanco C (2022) Living alone and suicide risk in the United States, 2008‒2019. *American Journal of Public Health* 112, 1774–1782. 36383944 10.2105/AJPH.2022.307080 PMC9670225

[ref31] Poudel-Tandukar K, Nanri A, Mizoue T, Matsushita Y, Takahashi Y, Noda M, Inoue M, Tsugane S and Japan Public Health Center-based Prospective Study Group (2011) Differences in suicide risk according to living arrangements in Japanese men and women – The Japan Public Health Center-based (JPHC) prospective study. *Journal of Affective Disorders* 131, 113–119. 21168916 10.1016/j.jad.2010.11.027

[ref32] Prentice RL, Pettinger M and Anderson GL (2005) Statistical issues arising in the Women’s Health Initiative. *Biometrics* 61, 899–911; discussion 911–41. 10.1111/j.0006-341X.2005.454_1.x 16401257

[ref33] Rehkopf DH and Buka SL (2006) The association between suicide and the socio-economic characteristics of geographical areas: A systematic review. *Psychological Medicine* 36, 145–157. 16420711 10.1017/S003329170500588X

[ref34] Sawada N, Nakaya T, Kashima S, Yorifuji T, Hanibuchi T, Charvat H, Yamaji T, Iwasaki M, Inoue M, Iso H and Tsugane S (2022) Long-term exposure to fine particle matter and all-cause mortality and cause-specific mortality in Japan: The JPHC Study. *BMC Public Health* 22, 466. 10.1186/s12889-022-12829-2 PMC8905772 35260115

[ref35] Schneider B, Grebner K, Schnabel A, Hampel H, Georgi K and Seidler A (2011) Impact of employment status and work-related factors on risk of completed suicide. A case-control psychological autopsy study. *Psychiatry Research* 190, 265–270. 21890214 10.1016/j.psychres.2011.07.037

[ref36] Shaw RJ (2022) Living alone and suicide risk: A complex problem requiring a whole population approach. *American Journal of Public Health* 112, 1699–1701. 36383942 10.2105/AJPH.2022.307138 PMC9670235

[ref37] Shaw RJ, Cullen B, Graham N, Lyall DM, Mackay D, Okolie C, Pearsall R, Ward J, John A and Smith DJ (2021) Living alone, loneliness and lack of emotional support as predictors of suicide and self-harm: A nine-year follow up of the UK Biobank cohort. *Journal of Affective Disorders* 279, 316–323. 33096330 10.1016/j.jad.2020.10.026 PMC7758739

[ref38] Shaw RJ, Harron KL, Pescarini JM, Pinto Junior EP, Allik M, Siroky AN, Campbell D, Dundas R, Ichihara MY, Leyland AH, Barreto ML and Katikireddi SV (2022) Biases arising from linked administrative data for epidemiological research: A conceptual framework from registration to analyses. *European Journal of Epidemiology* 37, 1215–1224. 36333542 10.1007/s10654-022-00934-w PMC9792414

[ref39] Shikimoto R, Nozaki S, Sawada N, Shimizu Y, Svensson T, Nakagawa A, Mimura M and Tsugane S (2022) Coping in mid- to late life and risk of mild cognitive impairment subtypes and dementia: A JPHC Saku Mental Health Study. *Journal of Alzheimer’s Disease* 90, 1085–1101. 10.3233/JAD-215712 PMC9741735 36213991

[ref40] Solmi M, Veronese N, Galvano D, Favaro A, Ostinelli EG, Noventa V, Favaretto E, Tudor F, Finessi M, Shin JI, Smith L, Koyanagi A, Cester A, Bolzetta F, Cotroneo A, Maggi S, Demurtas J, De Leo D and Trabucchi M (2020) Factors associated with loneliness: An umbrella review of observational studies. *Journal of Affective Disorders* 271, 131–138. 32479308 10.1016/j.jad.2020.03.075

[ref41] Stanley B, Martínez-Alés G, Gratch I, Rizk M, Galfalvy H, Choo T-H and Mann JJ (2021) Coping strategies that reduce suicidal ideation: An ecological momentary assessment study. *Journal of Psychiatric Research* 133, 32–37. 33307352 10.1016/j.jpsychires.2020.12.012 PMC8659118

[ref42] Stekhoven DJ and Bühlmann P (2012) MissForest—Non-parametric missing value imputation for mixed-type data. *Bioinformatics* 28, 112–118. 22039212 10.1093/bioinformatics/btr597

[ref43] Stickley A and Koyanagi A (2016) Loneliness, common mental disorders and suicidal behavior: Findings from a general population survey. *Journal of Affective Disorders* 197, 81–87. 26971125 10.1016/j.jad.2016.02.054

[ref44] Stickley A, Waldman K, Sumiyoshi T, Narita Z, Shirama A, Shin JI and Oh H (2021) Childhood physical neglect and psychotic experiences: Findings from the National Comorbidity Survey Replication. *Early Intervention in Psychiatry* 15, 256–262. 32048480 10.1111/eip.12932

[ref45] Stickley A, Waldman K, Ueda M, Koyanagi A, Sumiyoshi T, Narita Z, Inoue Y, DeVylder JE and Oh H (2020) Childhood neglect and suicidal behavior: Findings from the National Comorbidity Survey Replication. *Child Abuse and Neglect* 103, 104400. 10.1016/j.chiabu.2020.104400 32146267

[ref46] Tadmon D and Bearman PS (2023) Differential spatial-social accessibility to mental health care and suicide. *Proceedings of the National Academy of Sciences of the United States of America.* 120, e2301304120. 10.1073/pnas.2301304120 PMC10175830 37126686

[ref47] Tsugane S and Sawada N (2014) The JPHC study: Design and some findings on the typical Japanese diet. *Japanese Journal of Clinical Oncology* 44, 777–782. 25104790 10.1093/jjco/hyu096

[ref48] U.S. Census Bureau (2020) Older adults who are foreign born less likely to live alone than native born. https://www.census.gov/library/stories/2020/08/young-adults-most-likely-to-change-living-arrangements.html (accessed 15 May 2024).

[ref49] Vancampfort D, Hallgren M, Firth J, Rosenbaum S, Schuch FB, Mugisha J, Probst M, Van Damme T, Carvalho AF and Stubbs B (2018) Physical activity and suicidal ideation: A systematic review and meta-analysis. *Journal of Affective Disorders* 225, 438–448. 28858658 10.1016/j.jad.2017.08.070

[ref50] VanderWeele TJ (2019) Principles of confounder selection. *European Journal of Epidemiology* 34, 211–219. 30840181 10.1007/s10654-019-00494-6 PMC6447501

[ref51] VanderWeele TJ and Ding P (2017) Sensitivity analysis in observational research: Introducing the E-value. *Annals of Internal Medicine.* 167, 268–274. 28693043 10.7326/M16-2607

[ref52] Van Orden KA, Witte TK, Cukrowicz KC, Braithwaite SR, Selby EA and Joiner TE (2010) The interpersonal theory of suicide. *Psychological Review* 117, 575–600. 20438238 10.1037/a0018697 PMC3130348

[ref53] Willett WC, Howe GR and Kushi LH (1997) Adjustment for total energy intake in epidemiologic studies. *The American Journal of Clinical Nutrition* 65, 1220S-1228S; discussion 1229S-1231S. 10.1093/ajcn/65.4.1220S 9094926

[ref54] World Health Organization (1990) *International Statistical Classification of Diseases and Related Health Problems*, 1st edn. Geneva, Switzerland: World Health Organization.

[ref55] World Health Organization (2021a) Suicide. https://www.who.int/news-room/fact-sheets/detail/suicide (accessed 15 May 2024).

[ref56] World Health Organization (2021b). Suicide rates. https://www.who.int/data/gho/data/themes/mental-health/suicide-rates (accessed 18 April 2024).

[ref57] Wu VC-C, Chang S-H, Kuo C-F, Liu J-R, Chen S-W, Yeh Y-H, Luo S-F and See L-C (2018) Suicide death rates in patients with cardiovascular diseases – A 15-year nationwide cohort study in Taiwan. *Journal of Affective Disorders* 238, 187–193. 29885608 10.1016/j.jad.2018.05.046

[ref58] Yoshioka E, Hanley SJB, Sato Y and Saijo Y (2021) Geography of suicide in Japan: Spatial patterning and rural–urban differences. *Social Psychiatry & Psychiatric Epidemiology* 56, 731–746. 33159535 10.1007/s00127-020-01978-7 PMC8068717

[ref59] Zaorsky NG, Zhang Y, Tuanquin L, Bluethmann SM, Park HS and Chinchilli VM (2019) Suicide among cancer patients. *Nature Communications* 10, 207. 10.1038/s41467-018-08170-1 PMC6331593 30643135

