# Supplementary material for: Time-varying living arrangements and suicide death in the general population sample: 14-year causal survival analysis via pooled logistic regression
Source: Epidemiol Psychiatr Sci. 2024 May 23;33:e30. doi: 10.1017/S2045796024000325 (PMC11362678; doi:10.1017/S2045796024000325)
Supplement: Narita et al. supplementary material [file S2045796024000325sup001.docx]

Supplementary Appendix1. Model specifications for time-varying exposures, censoring during the follow-up, and inverse probability-weighted pooled logistic models

First, we calculated stabilized weights for the time-varying exposures (living arrangements at waves 1 and 2) before the start of follow-up (wave 2) to adjust for the covariates considered as potential confounders measured at waves 1 and 2. Wave 1 was defined as 1995–1999, depending on the timing of the survey conducted at each public health center, and wave 2 was defined as five years after wave 1, i.e., 2000–2004. The exposure probabilities with or without being conditional on the previous covariates were modeled by the logistic models.

(Model 1a) logit *P*(Living alone at wave 1) = *a* _wave 1,0_

(Model 1b) logit *P*(Living alone at wave 1| Covariates at wave 1)

= *b* _wave 1,0_ + *b* _wave 1,1_ Covariates at wave 1

(Model 2a) logit *P*(Living alone at wave 2| Living arrangements at wave 1)

= *a* _wave 2,0_ + *a* _wave 2,1_ Living alone at wave 1

(Model 2b) logit *P*(Living alone at wave 2| Living arrangements at wave 1,

Covariates at waves 1 and 2)

= *b* _wave 2,0_ + *b* _wave 2,1_ Living alone at wave 1

+ *b* _wave 2,2_ Covariates at wave 1 + *b* _wave 2,3_ Covariates at wave 2

We predicted the probabilities of living alone at waves 1 and 2 from each model based on the conditioning variables. We denote these predicted probabilities by *P*_1a_, *P*_1b_, *P*_2a_, and *P*_2b_, respectively. The stabilized weights for time-varying exposures were obtained as follows:

| Exposure status | Stabilized weight for exposure (SW^Living arrangements^) |
| --- | --- |
| Living alone both at waves 1 and 2 | [*P*_1a_/*P*_1b_][*P*_2a_/*P*_2b_] |
| Living alone at wave 1 but with someone at wave 2 | [*P*_1a_/*P*_1b_][(1 – *P*_2a_)/(1 – *P*_2b_)] |
| Living with someone at wave 1 but alone at wave 2 | [(1 – *P*_1a_)/(1 – *P*_1b_)][*P*_2a_/*P*_2b_] |
| Living with someone both at waves 1 and 2 | [(1 – *P*_1a_)/(1 – *P*_1b_)] [(1 – *P*_2a_)/(1 – *P*_2b_)] |

Next, we calculated stabilized weights for being uncensored during the 14-year follow-up period. The censoring probabilities during year *k* (*k* = 0, 1, …, 13) among participants who had been uncensored before that year were modeled by pooled logistic regression models:

(Model 3a) logit *P*(Censored at year *k*| Living arrangements at waves 1 and 2,

Uncensored before year *k*)

= *c*_0_ + *c*_1_ *k* + *c*_2_ *k*^2^ + *c*_3_ Living alone at wave 1

+ *c*_4_ Living alone at wave 2

(Model 3b) logit *P*(Censored at year *k*| Living arrangements at waves 1 and 2,

Covariates at waves 1 and 2, Uncensored before year *k*)

= *d*_0_ + *d*_1_ *k* + *d*_2_ *k*^2^ + *d*_3_ Living alone at wave 1

+ d4 Covariates at wave 1 + d5 Living alone at wave 2

+ d6 Covariates at wave 2

The stabilized weights adjusting for censoring before year *k* were calculated using the predicted censoring probabilities during year *k*, denoted by *P*_3a,_*_k_* and *P*_3b,_*_k_* from Models 3a and 3b, respectively:

SW*_k_*^Censoring^ = $\frac{\text{1 – }\text{P}\text{3a,0}}{\text{1 – }\text{P}\text{3b,0}}\times\frac{\text{1 – }\text{P}\text{3a,1}}{\text{1 – }\text{P}\text{3b,}\text{1}}\times\cdots\times\frac{\text{1 – }\text{P}\text{3a,}\text{k}}{\text{1 – }\text{P}\text{3b,}\text{k}}$

All the weights were truncated at the first and 99th percentiles before being multiplied by other weights.

Finally, we fit the pooled logistic model to the incidence probabilities of death during year *k* by weighting with time-varying stabilized weights SW^Living arrangements^ $\times$ SW*_k_*^Censoring^. The model includes the year, exposure groups, and their product terms to estimate the exposure group-specific survival curves:

logit *P*(Death at year *k*| Living arrangements at waves 1 and 2,

Living and uncensored before *k*)

= *e*_0_ + *e*_1_ *k* + *e*_2_ *k*^2^ + *e*_3_ Living alone at wave 1 + *e*_4_ Living alone at wave 2

+ *e*_5_ Living alone at wave 1 * Living alone at wave 2
+ *e*_6_ *k* * Living alone at wave 1 + *e*_7_ *k* * Living alone at wave 2
+ *e*_8_ *k* * Living alone at wave 1 * Living alone at wave 2

Note that the product terms between the quadratic term of year *k*^2^ and living arrangements were not included in the models due to the lack of meaningful coefficients in our exploratory analysis. The quadratic term *k*^2^ and the product terms of *k* and living arrangements were further excluded from the age-specific and gender-specific models due to the insufficient number of cases in each exposure group:

logit *P*(Death at year *k*| Living arrangements at waves 1 and 2,

Living and uncensored before *k*, Age)

= *e*_0,Age_ + *e*_1,Age_ *k* + *e*_2,Age_ Living alone at wave 1

+ *e*_3,Age_ Living alone at wave 2

+ *e*_4,Age_ Living alone at wave 1 * Living alone at wave 2

logit *P*(Death at year *k*| Living arrangements at waves 1 and 2,

Living and uncensored before *k*, Gender)

= *e*_0,Gender_ + *e*_1,Gender_ *k* + *e*_2,Gender_ Living alone at wave 1

+ *e*_3,Gender_ Living alone at wave 2

+ *e*_4,Gender_ Living alone at wave 1 * Living alone at wave 2

The time-dependent hazard ratios were estimated by combining the estimates of coefficients *e*_3_ to *e*_8_.

The cumulative incidence probabilities of death for each exposure group were estimated through the predicted probabilities of death during year *k*, *P*_Death_*_,k_*, from the fitted weighted pooled logistic model, calculated as:

Cumulative incidence probability at *k* in the exposure group

= 1 – (1 – *P*_Death_*_,_*_0_) (1 – *P*_Death_*_,_*_1_)… (1 – *P*_Death_*_,k_*).

The confidence intervals for the risk differences and ratios were separately obtained at each *k* (i.e., pointwise confidence intervals) using percentiles based on the 200 bootstrap samples.

Supplementary Figure 1

Flow diagram of study population selection.


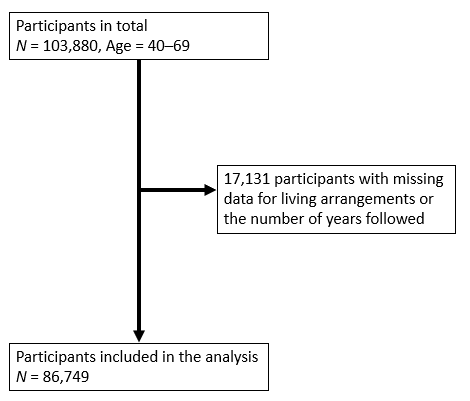


Supplementary Figure 2. Comparison of the Kaplan-Meier curve and the survival curve from unweighted pooled logistic regression for suicide death (A and B), non-suicide death (C and D), and all-cause mortality (E and F) in predicting a survival function.

**
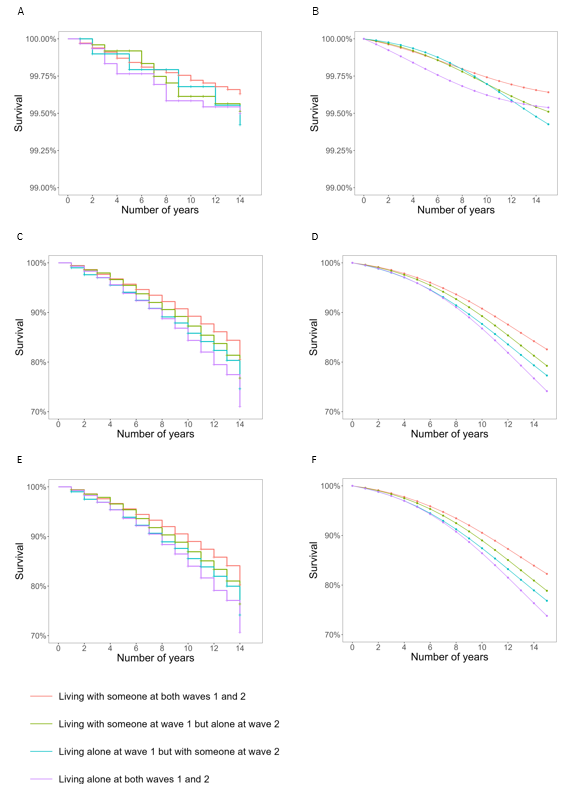
**

Supplementary Table 1. Sensitivity analysis: adjusting for the proxies of prior mental illness, social support, and coping.

|  |  |  | Suicide death | | | Non-suicide death | | | All-cause mortality | | |
| --- | --- | --- | --- | --- | --- | --- | --- | --- | --- | --- | --- |
| Living arrangements at wave 1 | Living arrangements at wave 2 | Person-years | Number of deaths | RD (95% bootstrap CI), % | RR (95% bootstrap CI) | Number of deaths | RD (95% bootstrap CI), % | RR (95% bootstrap CI) | Number of deaths | RD (95% bootstrap CI), % | RR (95% bootstrap CI) |
|  |  |  | Seven years after wave 2 (the midpoint of the follow-up period) | | | | | | | | |
| Living with someone | Living with someone | 620275 | 163 | 0.0 | 1.00 | 5214 | 0.0 | 1.00 | 5377 | 0.0 | 1.00 |
| Living with someone | Living alone | 19190 | 6 | 0.0  (-0.2, 0.2) | 1.08  (0.34, 1.97) | 198 | 1.0  (0.1, 1.9) | 1.20  (1.03, 1.39) | 204 | 1.0  (0.2, 2.0) | 1.19  (1.03, 1.40) |
| Living alone | Living with someone | 7605 | 2 | 0.0  (-0.2, 0.6) | 1.07  (2e-5, 3.94) | 91 | 0.6  (-1.4, 3.1) | 1.13  (0.72, 1.64) | 93 | 0.7  (-1.4, 3.0) | 1.13  (0.73, 1.58) |
| Living alone | Living alone | 23193 | 9 | 1.1  (0.3, 2.6) | 6.23  (2.07, 13.51) | 279 | 3.2  (1.8, 4.9) | 1.65  (1.37, 2.00) | 288 | 4.3  (2.6, 6.2) | 1.84  (1.51, 2.22) |
|  |  |  | 14 years after wave 2 (the end of the follow-up period) | | | | | | | | |
| Living with someone | Living with someone | 1100640 | 276 | 0.0 | 1.00 | 15365 | 0.0 | 1.00 | 15641 | 0.0 | 1.00 |
| Living with someone | Living alone | 33437 | 11 | 0.2  (-0.1, 0.5) | 1.44  (0.65, 2.32) | 563 | 3.1  (1.7, 4.9) | 1.22  (1.12, 1.34) | 574 | 3.2  (1.8, 4.9) | 1.23  (1.12, 1.34) |
| Living alone | Living with someone | 13196 | 5 | 0.6  (-0.3, 1.8) | 2.67  (0.22, 5.92) | 246 | 1.6  (-1.8, 5.4) | 1.11  (0.87, 1.38) | 251 | 2.1  (-1.2, 5.6) | 1.15  (0.92, 1.39) |
| Living alone | Living alone | 39780 | 14 | 1.4  (0.3, 3.1) | 4.60  (1.80, 9.41) | 852 | 8.0  (5.5, 10.7) | 1.57  (1.39, 1.76) | 866 | 9.0  (6.5, 11.6) | 1.63  (1.45, 1.80) |

Abbreviation: RD, risk difference; RR, risk ratio; CI, confidence interval; NA, not applicable.

Wave 1 was defined as the period from 1995 to 1999, depending on the timing of data collection at each public health center area, while wave 2 was defined as five years after wave 1, from 2000 to 2004.

The RD and RR were calculated based on cumulative incidence functions estimated by the inverse probability of exposure and censoring weighted pooled logistic regression accounting for covariates measured at waves 1 and 2 in the weights.

Age, gender, body mass index, smoking status, alcohol consumption, physical activity, employment status, sleep duration, history of cancer, history of cerebrovascular or cardiovascular disease, vegetable consumption, fruit consumption, fish consumption, meat consumption, and region at wave 1 were controlled for. Also, smoking status, employment status, sleep duration, history of cancer, history of cerebrovascular or cardiovascular disease, vegetable consumption, fruit consumption, fish consumption, and meat consumption at wave 2 were controlled for because these factors may be affected by living arrangements at wave 1 and also confound the association of living arrangements at wave 2 with suicide death, non-suicide death, and all-cause mortality. Moreover, the proxies of prior mental illness, social support, and coping were controlled for.

Supplementary Table 2. Age-stratified associations: participants aged 60 years or older.

|  |  |  | Suicide death | | | Non-suicide death | | | All-cause mortality | | |
| --- | --- | --- | --- | --- | --- | --- | --- | --- | --- | --- | --- |
| Living arrangements at wave 1 | Living arrangements at wave 2 | Person-years | Number of deaths | RD (95% bootstrap CI), % | RR (95% bootstrap CI) | Number of deaths | RD (95% bootstrap CI), % | RR (95% bootstrap CI) | Number of deaths | RD (95% bootstrap CI), % | RR (95% bootstrap CI) |
|  |  |  | Seven years after wave 2 (the midpoint of the follow-up period) | | | | | | | | |
| Living with someone | Living with someone | 90465 | 23 | 0.0 | 1.00 | 2140 | 0.0 | 1.00 | 2163 | 0.0 | 1.00 |
| Living with someone | Living alone | 5528 | 2 | 0.0  (-0.2, 0.1) | 0.84  (5e-7, 1.98) | 106 | -2.4  (-3.9, -0.9) | 0.85  (0.76, 0.95) | 108 | -2.4  (-3.9, -0.8) | 0.85  (0.77, 0.95) |
| Living alone | Living with someone | 1819 | 0 | NA | NA | 62 | 9.1  (3.6 17.1) | 1.57  (1.22, 2.09) | 62 | 9.1  (3.5, 17.1) | 1.56  (1.21, 2.07) |
| Living alone | Living alone | 8305 | 2 | 0.3  (-0.2, -0.1) | 2.48  (3e-7, 6.68) | 152 | -0.5  (-2.6, 1.9) | 0.97  (0.84, 1.12) | 154 | -0.3  (-2.7, 2.0) | 0.98  (0.84, 1.13) |
|  |  |  | 14 years after wave 2 (the end of the follow-up period) | | | | | | | | |
| Living with someone | Living with someone | 147020 | 39 | 0.0 | 1.00 | 6132 | 0.0 | 1.00 | 6171 | 0.0 | 1.00 |
| Living with someone | Living alone | 9142 | 3 | -0.1  (-0.4, 0.3) | 0.84  (5e-7, 1.98) | 313 | -5.5  (-9.1, -1.9) | 0.88  (0.80, 0.96) | 316 | -5.5  (-8.9, -1.9) | 0.88  (0.81, 0.96) |
| Living alone | Living with someone | 2906 | 0 | NA | NA | 149 | 17.7  (7.6, 29.3) | 1.39  (1.16, 1.66) | 149 | 17.3  (7.3, 29.0) | 1.38  (1.16, 1.65) |
| Living alone | Living alone | 13551 | 4 | 0.5  (-0.4, 1.9) | 2.48  (3e-7, 6.68) | 514 | -1.1  (-6.0, 4.0) | 0.98  (0.87, 1.09) | 518 | -0.8  (-6.1, 4.3) | 0.98  (0.87, 1.10) |

Abbreviation: RD, risk difference; RR, risk ratio; CI, confidence interval; NA, not applicable.

Wave 1 was defined as the period from 1995 to 1999, depending on the timing of data collection at each public health center, while wave 2 was defined as five years after wave 1, from 2000 to 2004.

The RD and RR were calculated based on cumulative incidence functions estimated by the inverse probability of exposure and censoring weighted pooled logistic regression accounting for covariates measured at waves 1 and 2 in the weights.

Age, gender, body mass index, smoking status, alcohol consumption, physical activity, employment status, sleep duration, history of cancer, history of cerebrovascular or cardiovascular disease, vegetable consumption, fruit consumption, fish consumption, meat consumption, and region at wave 1 were controlled for. Also, smoking status, employment status, sleep duration, history of cancer, history of cerebrovascular or cardiovascular disease, vegetable consumption, fruit consumption, fish consumption, and meat consumption at wave 2 were controlled for because these factors may be affected by living arrangements at wave 1 and also confound the association of living arrangements at wave 2 with suicide death, non-suicide death, and all-cause mortality.

Supplementary Table 3. Gender-stratified associations: participants aged 59 years or younger.

|  |  |  | Suicide death | | | Non-suicide death | | | All-cause mortality | | |
| --- | --- | --- | --- | --- | --- | --- | --- | --- | --- | --- | --- |
| Living arrangements at wave 1 | Living arrangements at wave 2 | Person-years | Number of deaths | RD (95% bootstrap CI), % | RR (95% bootstrap CI) | Number of deaths | RD (95% bootstrap CI), % | RR (95% bootstrap CI) | Number of deaths | RD (95% bootstrap CI), % | RR (95% bootstrap CI) |
|  |  |  | Seven years after wave 2 (the midpoint of the follow-up period) | | | | | | | | |
| Living with someone | Living with someone | 529810 | 140 | 0.0 | 1.00 | 3074 | 0.0 | 1.00 | 3214 | 0.0 | 1.00 |
| Living with someone | Living alone | 13662 | 4 | 0.1  (0.0, 0.3) | 1.65  (0.75, 2.73) | 92 | 0.2  (-0.3, 0.7) | 1.05  (0.90, 1.19) | 96 | 0.3  (-0.3, 0.7) | 1.07  (0.91, 1.20) |
| Living alone | Living with someone | 5786 | 2 | 0.3  (-0.1, 0.8) | 2.49  (0.19, 5.74) | 29 | 0.3  (-0.8, 1.5) | 1.08  (0.77, 1.44) | 31 | 0.5  (-0.7, 1.8) | 1.12  (0.81, 1.48) |
| Living alone | Living alone | 14888 | 7 | 0.4  (0.0, 0.8) | 3.07  (1.04, 5.80) | 127 | 1.9  (1.0, 2.6) | 1.51  (1.28, 1.75) | 134 | 2.1  (1.1, 3.0) | 1.56  (1.31, 1.81) |
|  |  |  | 14 years after wave 2 (the end of the follow-up period) | | | | | | | | |
| Living with someone | Living with someone | 953620 | 237 | 0.0 | 1.00 | 9233 | 0.0 | 1.00 | 9470 | 0.0 | 1.00 |
| Living with someone | Living alone | 24295 | 8 | 0.2  (-0.1, 0.6) | 1.65  (0.75, 2.72) | 250 | 0.5  (-1.0, 1.8) | 1.05  (0.90, 1.18) | 258 | 0.7  (-0.9, 2.0) | 1.07  (0.91, 1.19) |
| Living alone | Living with someone | 10290 | 5 | 0.5  (-0.3, 1.6) | 2.49  (0.19, 5.72) | 97 | 0.8  (-2.3, 4.2) | 1.08  (0.78, 1.42) | 102 | 1.2  (-1.9, 4.8) | 1.12  (0.82, 1.46) |
| Living alone | Living alone | 26229 | 10 | 0.7  (0.1, 1.6) | 3.07  (1.04, 5.79) | 338 | 4.9  (2.7, 7.1) | 1.48  (1.27, 1.70) | 348 | 5.5  (3.1, 7.9) | 1.53  (1.29, 1.76) |

Abbreviation: RD, risk difference; RR, risk ratio; CI, confidence interval; NA, not applicable.

Wave 1 was defined as the period from 1995 to 1999, depending on the timing of data collection at each public health center, while wave 2 was defined as five years after wave 1, from 2000 to 2004.

The RD and RR were calculated based on cumulative incidence functions estimated by the inverse probability of exposure and censoring weighted pooled logistic regression accounting for covariates measured at waves 1 and 2 in the weights.

Age, gender, body mass index, smoking status, alcohol consumption, physical activity, employment status, sleep duration, history of cancer, history of cerebrovascular or cardiovascular disease, vegetable consumption, fruit consumption, fish consumption, meat consumption, and region at wave 1 were controlled for. Also, smoking status, employment status, sleep duration, history of cancer, history of cerebrovascular or cardiovascular disease, vegetable consumption, fruit consumption, fish consumption, and meat consumption at wave 2 were controlled for because these factors may be affected by living arrangements at wave 1 and also confound the association of living arrangements at wave 2 with suicide death, non-suicide death, and all-cause mortality.

Supplementary Table 4. Gender-stratified associations: female participants.

|  |  |  | Suicide death | | | Non-suicide death | | | All-cause mortality | | |
| --- | --- | --- | --- | --- | --- | --- | --- | --- | --- | --- | --- |
| Living arrangements at wave 1 | Living arrangements at wave 2 | Person-years | Number of deaths | RD (95% bootstrap CI), % | RR (95% bootstrap CI) | Number of deaths | RD (95% bootstrap CI), % | RR (95% bootstrap CI) | Number of deaths | RD (95% bootstrap CI), % | RR (95% bootstrap CI) |
|  |  |  | Seven years after wave 2 (the midpoint of the follow-up period) | | | | | | | | |
| Living with someone | Living with someone | 333368 | 50 | 0.0 | 1.00 | 1726 | 0.0 | 1.00 | 1776 | 0.0 | 1.00 |
| Living with someone | Living alone | 13690 | 5 | 0.1  (0.0, 0.3) | 2.11  (0.59, 4.06) | 99 | 1.0  (0.6, 1.5) | 1.33  (1.18, 1.50) | 104 | 1.1  (0.6, 1.6) | 1.34  (1.19, 1.51) |
| Living alone | Living with someone | 5019 | 2 | 0.5  (0.0, 1.6) | 5.71  (0.05, 15.92) | 43 | 0.4  (-0.1 1.6) | 1.13  (0.81, 1.50) | 45 | 0.7  (-0.3, 1.9) | 1.22  (0.89, 1.61) |
| Living alone | Living alone | 17259 | 0 | NA | NA | 175 | 2.1  (1.2, 3.1) | 1.66  (1.38, 1.97) | 175 | 2.0  (1.1, 3.1) | 1.62  (1.35, 1.93) |
|  |  |  | 14 years after wave 2 (the end of the follow-up period) | | | | | | | | |
| Living with someone | Living with someone | 600998 | 84 | 0.0 | 1.00 | 5739 | 0.0 | 1.00 | 5823 | 0.0 | 1.00 |
| Living with someone | Living alone | 24248 | 8 | 0.2  (0.0, 0.6) | 2.11  (0.59, 4.05) | 322 | 3.0  (1.6, 4.5) | 1.31  (1.17, 1.47) | 330 | 3.2  (1.8, 4.7) | 1.33  (1.18, 1.48) |
| Living alone | Living with someone | 8824 | 4 | 1.0  (-0.2, 2.8) | 5.70  (0.05, 15.80) | 148 | 1.2  (-1.8, 4.6) | 1.12  (0.82, 1.47) | 152 | 2.1  (-1.0, 5.6) | 1.21  (0.90, 1.58) |
| Living alone | Living alone | 29887 | 3 | -0.2  (-0.2, -0.2) | 0.02  (9e-7, 0.06) | 573 | 6.0  (3.5, 8.8) | 1.62  (1.36, 1.90) | 576 | 5.8  (3.2, 8.6) | 1.59  (1.33, 1.87) |

Abbreviation: RD, risk difference; RR, risk ratio; CI, confidence interval; NA, not applicable.

Wave 1 was defined as the period from 1995 to 1999, depending on the timing of data collection at each public health center, while wave 2 was defined as five years after wave 1, from 2000 to 2004.

The RD and RR were calculated based on cumulative incidence functions estimated by the inverse probability of exposure and censoring weighted pooled logistic regression accounting for covariates measured at waves 1 and 2 in the weights.

Age, gender, body mass index, smoking status, alcohol consumption, physical activity, employment status, sleep duration, history of cancer, history of cerebrovascular or cardiovascular disease, vegetable consumption, fruit consumption, fish consumption, meat consumption, and region at wave 1 were controlled for. Also, smoking status, employment status, sleep duration, history of cancer, history of cerebrovascular or cardiovascular disease, vegetable consumption, fruit consumption, fish consumption, and meat consumption at wave 2 were controlled for because these factors may be affected by living arrangements at wave 1 and also confound the association of living arrangements at wave 2 with suicide death, non-suicide death, and all-cause mortality.

Supplementary Table 5. Gender-stratified associations: male participants.

|  |  |  | Suicide death | | | Non-suicide death | | | All-cause mortality | | |
| --- | --- | --- | --- | --- | --- | --- | --- | --- | --- | --- | --- |
| Living arrangements at wave 1 | Living arrangements at wave 2 | Person-years | Number of deaths | RD (95% bootstrap CI), % | RR (95% bootstrap CI) | Number of deaths | RD (95% bootstrap CI), % | RR (95% bootstrap CI) | Number of deaths | RD (95% bootstrap CI), % | RR (95% bootstrap CI) |
|  |  |  | Seven years after wave 2 (the midpoint of the follow-up period) | | | | | | | | |
| Living with someone | Living with someone | 286907 | 113 | 0.0 | 1.00 | 3488 | 0.0 | 1.00 | 3601 | 0.0 | 1.00 |
| Living with someone | Living alone | 5500 | 1 | 0.0  (-0.4, 0.7) | 1.04  (1e-6, 3.04) | 99 | 3.6  (2.0, 5.0) | 1.48  (1.27, 1.67) | 100 | 3.6  (0.2, 5.1) | 1.46  (1.26, 1.65) |
| Living alone | Living with someone | 2586 | 0 | NA | NA | 48 | 2.0  (-0.8, 5.3) | 1.26  (0.89, 1.70) | 48 | 2.0  (-0.7, 5.3) | 1.25  (0.91, 1.67) |
| Living alone | Living alone | 5934 | 9 | 0.5  (-0.2, 1.4) | 2.42  (0.59, 4.54) | 104 | 3.4  (1.6, 5.4) | 1.46  (1.21, 1.72) | 113 | 3.8  (1.7, 5.7) | 1.49  (1.21, 1.74) |
|  |  |  | 14 years after wave 2 (the end of the follow-up period) | | | | | | | | |
| Living with someone | Living with someone | 499642 | 192 | 0.0 | 1.00 | 9626 | 0.0 | 1.00 | 9818 | 0.0 | 1.00 |
| Living with someone | Living alone | 9189 | 3 | 0.0  (-0.7, 1.3) | 1.04  (1e-6, 3.03) | 241 | 8.6  (5.0, 11.9) | 1.42  (1.24, 1.59) | 244 | 8.6  (4.8, 11.9) | 1.41  (1.23, 1.57) |
| Living alone | Living with someone | 4372 | 1 | -0.1  (-0.7, 1.2) | 0.88  (1e-6, 3.18) | 98 | 4.8  (-2.1, 12.4) | 1.24  (0.90, 1.61) | 99 | 4.7  (-1.8, 12.2) | 1.22  (0.92, 1.59) |
| Living alone | Living alone | 9893 | 11 | 0.9  (-0.3, 2.5) | 2.42  (0.60, 4.51) | 279 | 8.3  (3.9, 12.8) | 1.41  (1.19, 1.63) | 290 | 8.9  (4.1, 13.2) | 1.43  (1.19, 1.64) |

Abbreviation: RD, risk difference; RR, risk ratio; CI, confidence interval; NA, not applicable.

Wave 1 was defined as the period from 1995 to 1999, depending on the timing of data collection at each public health center, while wave 2 was defined as five years after wave 1, from 2000 to 2004.

The RD and RR were calculated based on cumulative incidence functions estimated by the inverse probability of exposure and censoring weighted pooled logistic regression accounting for covariates measured at waves 1 and 2 in the weights.

Age, gender, body mass index, smoking status, alcohol consumption, physical activity, employment status, sleep duration, history of cancer, history of cerebrovascular or cardiovascular disease, vegetable consumption, fruit consumption, fish consumption, meat consumption, and region at wave 1 were controlled for. Also, smoking status, employment status, sleep duration, history of cancer, history of cerebrovascular or cardiovascular disease, vegetable consumption, fruit consumption, fish consumption, and meat consumption at wave 2 were controlled for because these factors may be affected by living arrangements at wave 1 and also confound the association of living arrangements at wave 2 with suicide death, non-suicide death, and all-cause mortality.
